# Statistical analysis of the university sustainability in the higher education institution a case study from the Khyber Pakhtunkhwa province in Pakistan

**DOI:** 10.1016/j.heliyon.2023.e16230

**Published:** 2023-05-12

**Authors:** Naseha Wafa Qammar, Zohaib Ur Rehman Afridi, Shamaima Wafa Qammar

**Affiliations:** a Department of Mathematics, Kaunas University of Technology, LT-51368 Kaunas, Lithuania; b Department of Energy Management and Sustainability, US, Pakistan; c Centre for Advanced Studies in Energy, University of Engineering and Technology Peshawar, Pakistan; d Civil Engineering Department, University of Engineering & Technology, Peshawar, Pakistan

**Keywords:** Sustainability, Higher education institute, Students and faculty, Pakistan

## Abstract

Educational institutions can incorporate the idea of sustainability at the grass root level for any society. This study is part of an effort to get insight into the campus sustainability in one of the Higher Education Institution (HEI) in the Khyber Pakhtunkhwa region of Pakistan. Aim is to investigation university students' and faculty members insight regarding sustainability. Thus, questionnaire-based survey followed by statistical inference was conducted for the potential outcomes. The questionnaire is comprised of 24 questions, 05 of which are on demographics and the remaining 19 are about sustainability. The sustainability related questions focused mostly on the respondents' knowledge, understanding, and interest in sustainability. A handful of the other questions in the questionnaire were tailored to the university input to achieve sustainability. The dataset is manipulated with basic statistical and computational approaches, and the results are analyzed using mean values. The mean values are further classified into flag values of 0 and 1. Flag value 1 indicates a good marker of the received response, while flag value 0 indicates the least amount of information included in responses. The results show that respondents' knowledge, awareness, interest, and engagement in sustainability are significantly sufficient, as we obtained a flag value of 1 for all questions about sustainability. The study's findings, on the other hand, indicated that the institution is lagging in terms of supporting, disseminating, and implementing campus-wide sustainability-related activities. This study is one of the first initiatives to provide a baseline dataset and substantial information to go a step further in achieving the bottom-line target of being and acting sustainable in the HEI.

## Highlights

• Descriptive analysis of the university sustainability in a developing country.
• Knowledge, understanding and implementation of sustainability inside the HEI.
• HEI inclusion in the sustainability related platforms.

## 1 Introduction

Understanding, achieving, and maintaining sustainability has become increasingly important in recent years, as seen by the severe and irreversible climatic changes occurring throughout the globe. These widespread and escalating climate shifts are bizarre and have alarmed life on Earth for future generations. The climatic occurrences are thought-provoking and are enough to drive us to ponder alternative solutions for preserving natural resources— this is where the concept of sustainability comes into play [1]. The intuition for the solution has been witnessed over the last few years, which is why we may see the industry [2,3], academia [4], transportation [5], business [6], agricultural [7], domestic [8], and other sectors playing a vital part in comprehending, accomplishing, and maintaining sustainability.

Likewise, Higher Education Institutions (HEI) have also been in the forefront of promoting and incorporating sustainability initiatives. Educational institutions serve as a promotional arena for expanding sustainability awareness and also for assessing its impact before and after the sustainability activities [9]. According to one of the Baltic studies, higher educational institutions have a significant effect on society and may play an essential part in ensuring sustainability [10]. The role of educational institutions in sustainability is not only limited to the participating bodies (students, faculty, staff) inside the institute but the university as a whole functions as one organized unit and can have an impact on the region [4]. One of the studies from United States has targeted HEI's for embracing and disseminating sustainable practices, for instance in one study the educational networks are regarded as the best sustainable measures to be implemented [11]. The widespread spectrum of the impact of the sustainability studies ensures a promising platform in disseminating the sustainability within a region. Understanding, comprehending, acting, and adapting to sustainability in HEI's is a critical challenge that must be addressed from a variety of perspectives. For example, when we speak and think about the sustainability in the university, several questions may arise in the minds, such as:

What is sustainability inside the university? Who is responsible for ensuring the university sustainability practices? How much interest is found in the university students as well as faculty about the sustainability? Do the students have an idea about the sustainability? What are the approaches the HEI's are adapting to achieve the sustainability? What are the practical level implementations of sustainability practices in universities, etc?

Extensive research has been conducted globally to answer the aforementioned and many other pertinent questions about sustainability inside the HEI's. Comprehending the definition of sustainability and recognizing the need of implementing sustainability practices in the university campuses is crucial [12]. University campuses require active efforts to ensure that students recognize and comprehend the significance of sustainability. Not only that, but the institution's job is also to disseminate the knowledge of sustainability across the campus. The sustainability practices inside the HEI's is divided into multidimensional areas such as the behavior of students and faculty towards water, land and energy use, disposal and recycling activities, environmental issues, the implementation of curricula, university programs, etc. [13]. We will discuss about a few case studies where HEI's have addressed the university sustainability initiatives from diverse perceptive such as understanding, interest, implementation, commitments towards being sustainable. According to one sustainability case study carried out at Alabama and Huawai College on students' perspectives and awareness of sustainability, it was reported that students are more interested about reusing waste materials and addressing environmental concerns. It is also noted that, while students have indicated an interest in implementing sustainable practices within the university, there appears to be a gap between their commitment and action [11]. According to another case study from the University of Aalborg, it was reported that students are concerned about the environmental problems. It is also anticipated that the university's commitment will play a significant role in initiating sustainable practices inside the university. In the same work, it is mentioned that the inclusion of the problem-based learning into the educational system will help to attain sustainability at university campus [14]. In these discussions from various HEIs, there has been a comparable proportion of insight where university students appear to be concerned, intelligible, and interested in sustainable practices.

We will now focus our attention to the case studies which investigated the community participation in sustainability practices [15]. However, before we go any further, we'd want to highlight a very captivating example from a case study in which the university campus is analogously regarded as the “factory model” [16]. In this model, the “inputs” are the materials, transport and electricity, etc. The intermediate products include students, academics, papers, scientific materials, faculty, staff etc, and the outputs are environmental concerns like pollutants and chemicals. In the factory model, the output is entirely influenced by the input and the intermediate products, meaning that the changing any of the input and intermediate products can completely impact the results on the environment. However, we will focus our attention on the intermediate products, which are also the mainstay of our study. If students' interests, engagement, understanding, and commitment to sustainability approaches lag, the output; meaning environmental problems will increase [16]. A case study by the University of Toronto in Mississauga (UTM) has also taken an essential approach to address and emphasize the fact that the students engagement in campus sustainability is one of the important parameter to attain sustainability [17].

Other crucial variables that must be addressed in order to achieve sustainability can be stretched to various communications channels [[18], [19], [20]]. For example, organizing seminars and conferences, setting up awareness-raising programs, promoting sustainability initiatives, setting up a curriculum can address the issue of barriers to green campus thinking [21]. There is no way to fully attain the sustainability inside the HEI if there is a lack of data access and information [22]. According to National University of Malaysia's sustainability structure reports, educational institutions serve as a model for the implementation of sustainability-related programs [23]. In order to expand the concept of campus sustainability, students and faculty must be made aware of sustainability prior to its implementation. The same argument was corroborated by one of the research studies, which concluded that students' understanding of campus sustainability is a crucial component prior to the implementation [24].

When we look at and examine the study approaches used by various HEIs around the globe, we can see that there is diversity in the study approach employed by the researchers, and it varies from one HEI to another HEI and may address one or many of the aforementioned factors to address sustainability within the HEI's. However, the bottom line in all of these research studies remains the same “the single goal of being and acting sustainably” to achieve one or many of the SGDs [25].

With the definite goal of addressing sustainability in HEIs, developing countries are also making significant efforts to understand, implement, and adapt sustainability. For instance, in a few case studies from Pakistan, curriculum is regarded as one of the important factors of sustainability [26,27]. Another case had regarded the entrepreneurship as one of the important driver for attaining sustainability [28]. A few studies highlighted the fact that there is least interest by the students and faculty in all areas of HEI's which may including the; curriculum, research, stakeholder's engagement and governance [28]. Overall, the statics show that the education sector is the most interactive platform for building sustainability awareness and for launching different sustainability programs [29].

Since the notion of HEI sustainability is broad, overlapping, and dynamic in terms of various aspects such as curriculum, interests, awareness, practical implementation, and so on. This study is part of an effort to identify the numerous factors relating sustainability in HEI at one of the universities in the KP region of Pakistan. Looking at the content for the literature review for the sustainability in HEI in Pakistan, it is noticeable that only in recent years there has been a growing interest in delving deeply into sustainability studies in HEI (- the synopsis of the extensive literature is beyond the scope of this study; we merely wanted to make it obvious that the literature content has only recently begun to emerge in the sphere of research on sustainability in Pakistan's HEI). Therefore, it is hypothesized that there is still a need to delve further into the area of sustainability in the HEI's in Pakistan, and hence this research study is one of several attempts to accomplish the same objective. The major goal is to analyze the numerous sustainability related questions from university students and faculty members and statistically analyze them to come up with the inclusion of the current sustainability scenario inside the university. Despite the study's simple and descriptive approach, the study analysis provides solid foundations for evaluating the sustainability overview of university in the KP province in Pakistan. The study analysis will highlight the significance of understanding and adopting sustainable practices among the university community, which includes students, faculty, administration, and support staff. Overall, HEIs are making significant contributions in achieving sustainability, and this research is one of the avenues via which sustainability practices may be comprehended, recognized and implemented in the universities in Pakistan. The collaborative contribution of HEI's may lead to the accomplishment of the United Nations 2030 Agenda for Sustainable Development and the accompanying Sustainable Development Goals (SDGs).

## 2 Methodology

### 2.1 Ethics statement

This survey questionnaire complies with the ethical review standards, and all responses are entirely confidential. The City University of Science and Information Technology's Institutional Review Board has granted authorization for conducting the survey on campus. All participants in the research provided informed consent. The respondent's right to privacy is respected, and all applicable data protection rules are observed.

### 2.2 Participants

The participants in this research study are the university students, faculty members, and staff members. According to the university's current statistics, the total number of active students is more than 1500, the number of faculty members is 104, and the student-to-teacher ratio is 15:1. There are a total of ten departments which includes the engineering, non-engineering, technology, science and the arts. The details upon the demographics of the university participants can be seen in Table 1 (for faculty) and Table 3 (for students).

**Table 1:** Demographics of the study for the faculty and administrative staff in the university.

Variables/Parameters	Statistical Analysis				
	Minimum	Maximum	Mean	Standard deviation	Range
**Gender** Gender of the respondent 1. Male 2. Female	1	2	1.24	0.43	1
**Position** Respondent position in the university? 1. Faculty member 2. Administration/Staff 3. Student	1	2	1.30	0.47	1
**Department** 1. Civil Engineering 2. Electrical Engineering 3. Architecture Department 4. English Department 5. Mathematics Department 6. Management Science 7. Health Sciences Department 8. Administration 9. Computer Science 10. Software Engineering	1	9	3.72	2.65	8
**Semester** Current semester of the respondent? (**for students only**)	–	–	–	–	–
**Highest Education** 1. Bachelor 2. Master 3. Doctoral 4. Post-doctoral	1	3	1.78	0.59	2

**Table 2:** Statistical analysis of the sustainability related questions inquired from the faculty and staff.

Question	Question Type	Question Label	Statistical analysis					
			Min	Max	Mean	Standard deviation	Range	Flag values
On a scale of 0–10 identify your understanding level about the meaning of sustainability?	Scale based	$S_{1}$	0	10	7.17	2.49	10	1
By sustainability you mean? Please choose only ONE option. 1. Increase in Population 2. Decrease in Population 3. Increase in Energy demand 4. Decrease in Energy demand 5. Did not answer 6. Did not understand	Option based	$O_{1}$	1	5	3.37	1.07	4	–
In your opinion which factor(s) is (are) the most important for Sustainability? 1. Social aspect 2. Environmental aspect 3. Economic aspect 4. All of them	Option based	$O_{2}$	1	4	3.71	0.88	3	–
Non- engineering fields can contribute to achieve sustainability. On a scale of 0–10 mark your agreement level?	Scale based	$S_{2}$	0	10	7.17	2.35	10	1
On a scale of 0–10 mark your knowledge level about “Green buildings”?	Scale based	$S_{3}$	0	10	6.00	2.31	10	1
To make City University a green campus which of the following can be the most important aspect(s)? 1. Arranging seminar/Workshops relating Sustainability 2. Initiating practices of Reuse, Reduce and Recycle of waste 3. Allocation of Incentives for the departments initiating the Sustainability practices 4. Both 1 & 2 5. Both 2 & 3 6. All of them	Option based	$O_{3}$	1	6	2.10	0.85	5	–
How much difference do you think can be made by the individual practicing the Sustainability? On a scale of 0–10.	Scale based	$S_{4}$	0	10	6.43	2.23	10	1
Data access for advancing in the field of sustainability is not important. On a scale of 0–10 mark your opinion in agreeing the statement?	Scale based	$S_{5}$	0	10	4.15	2.62	10	0
On a scale of 0–10 mark your knowledge about the current ranking of Pakistan in the Global Climate Risk Index list?	Scale based	$S_{6}$	0	10	4.54	2.80	10	0
Recycle, reduce and reuse practices can improve economy and achieve sustainability? On a scale of 0–10 mark your opinion in agreeing the statement?	Scale based	$S_{7}$	1	10	7.87	2.26	9	1
On a scale of 0–10 mark your participation interest in the seminars, conferences, workshops relating sustainability initiatives?	Scale based	$S_{8}$	0	10	7.14	3.01	10	1
On a scale of 0–10 mark your interest level in reading and (or) listening to the news, stories involving environment and (or) sustainability?	Scale based	$S_{9}$	3	10	7.02	2.12	7	1
How much you are willing to learn more about Sustainability?	Scale based	$S_{10}$	2	10	8.32	1.83	8	1
On a scale of 0–10, mark your preference level for job, if income is not a factor, would you prefer a job related to sustainable development over other jobs?	Scale based	$S_{11}$	0	10	6.87	2.50	10	1
Wastewater should be recycled and sold to local community on subsidized rates. On a scale of 0–10 mark your agreement level with the argument?	Scale based	$S_{12}$	1	10	7.70	2.14	9	1
How many seminars, conferences and other activities are arranged by City University relating Sustainability?	Scale based	$S_{13}$	0	10	3.87	3.05	10	0
Are you aware how many research-based activities/papers City University has published relating Sustainability till date?	Scale based	$S_{14}$	0	10	2.65	3.29	10	0
On a scale of 0–10 identify the university approach in offering any specialization or training courses regarding sustainability in the campus?	Scale based	$S_{15}$	0	10	3.22	3.18	10	0
In your opinion, how to achieve the goal to have a sustainable campus? 1. Students' intake reduction 2. Green building certification 3. Introducing Curriculum for Sustainability 4. Both 2 & 3 5. Did not answer 6. All of them	Option based	$O_{4}$	1	6	2.93	0.95	5	–

**Table 3:** Demographics of the study for the students responses in the university.

Variables/Parameters	Statistical Analysis				
	Minimum	Maximum	Mean	Standard deviation	Range
**Gender** Gender of the respondent 1. Male 2. Female	1	2	1.118	0.324	1
**Position** Respondent position in the university? 1. Faculty member 2. Administration Staff 3. Student	1	3	2.976	0.216	2
**Department** 1. Civil Engineering 2. Electrical Engineering 3. Architecture Department 4. English Department 5. Mathematics Department 6. Management Science 7. Health Sciences Department 8. Administration 9. Computer Science 10. Software Engineering	1	10	2.847	2.152	9
**Semester** Current semester of the respondent? (**for students only**)	1	8	5.22	1.88	7
**Highest Education** 1. Bachelor 2. Master 3. Doctoral 4. Post-doctoral	1	3	1.082	0.352	2

### 2.3 Structure of research questionnaire, distribution, collection, and computations

The survey questionnaire consists of a total of k = 24 queries, of which 09 are multi-choice questions and 15 are scale-based questions from 0 to 10 (where 0 represents a low scale and 10 represents a high scale). The questionnaire is also comprised of the closed ended questions. The study responses are categorized and analyzed in two ways.

1. Campus faculty and staff response.
2. Campus students' response.

The main idea of categorization and analyzing the questionnaire in two domains is because the experience and knowledge level of the university faculty and staff members will absolutely differ from those of the university students’ responses. Details of the questions inquired are listed in Table 2 and Table 4. The questionnaire was distributed by hand among the various departments of the university and a week time is allocated to recollect the responses from all the departments. A total of (n = 131) questionnaires are received out of which (n = 85) responses are from the students and the remaining (n = 46) responses are from the faculty and the staff members of the university. With a margin of error of 5% and a confidence level of 90% a total of (n = 131) are analyzed using the computational and statistical techniques. The scale-based questions are analyzed based on the answers received for scale-based questions. Out of the k = 24 questions, the initial five questions are about the demographics of the study, for instance the gender, education level, position, semester (only for students) and highest education of the respondents. The remaining k = 19 questions are about the sustainability in the HEI.

**Table 4:** Statistical analysis of the sustainability related questions inquired from students.

Question	Question Type	Question Label	Statistical analysis					
			Min	Max	Mean	Standard deviation	Range	Flag values
On a scale of 0–10 identify your understanding level about the meaning of sustainability?	Scale based	$S_{1}$	0	10	6.07	2.74	10	1
By sustainability you mean? Please choose only ONE option. 1. Increase in Population 2. Decrease in Population 3. Increase in Energy demand 4. Decrease in Energy demand 5. Did not answer 6. Did not understand	Option based	$O_{1}$	1	6	2.68	1.36	5	–
In your opinion which factor(s) is (are) the most important for Sustainability? 1. Social aspect 2. Environmental aspect 3. Economic aspect 4. All of them	Option based	$O_{2}$	2	4	3.49	0.69	2	–
Non- engineering fields can contribute to achieve sustainability. On a scale of 0–10 mark your agreement level?	Scale based	$S_{2}$	0	10	6.07	2.60	10	1
On a scale of 0–10 mark your knowledge level about “Green buildings”?	Scale based	$S_{3}$	0	10	7.14	2.36	10	1
To make City University a Green campus which of the following can be the most important aspect(s)? 1. Arranging seminar/Workshops relating Sustainability 2. Initiating practices of Reuse, Reduce and Recycle of waste 3. Allocation of Incentives for the departments initiating the Sustainability practices 4. Both 1 & 2 5. Both 2 & 3 6. All of them	Option based	$O_{3}$	1	6	1.63	1.32	5	–
How much difference do you think can be made by the individual practicing the Sustainability? On a scale of 0–10.	Scale based	$S_{4}$	1	10	6.67	2.40	9	1
Data access for advancing in the field of sustainability is not important. On a scale of 0–10 mark your opinion in agreeing the statement?	Scale based	$S_{5}$	0	10	5.27	2.83	10	1
On a scale of 0–10 mark your knowledge about the current ranking of Pakistan in the Global Climate Risk Index list?	Scale based	$S_{6}$	0	10	6.58	2.66	10	1
Recycle, reduce and reuse practices can improve economy and achieve sustainability? On a scale of 0–10 mark your opinion in agreeing the statement?	Scale based	$S_{7}$	1	10	7.87	2.26	9	1
On a scale of 0–10 mark your participation interest in the seminars, conferences, workshops relating sustainability initiatives?	Scale based	$S_{8}$	0	10	6.48	3.01	10	1
On a scale of 0–10 mark your interest level in reading and (or) listening to the news, stories involving environment and (or) sustainability?	Scale based	$S_{9}$	0	10	7.05	2.36	10	1
How much you are willing to learn more about Sustainability?	Scale based	$S_{10}$	0	10	7.35	2.67	10	1
On a scale of 0–10, mark your preference level for job, if income is not a factor, would you prefer a job related to sustainable development over other jobs?	Scale based	$S_{11}$	0	10	6.48	2.45	10	1
Wastewater should be recycled and sold to local community on subsidized rates.On a scale of 0–10 mark your agreement level with the argument?	Scale based	$S_{12}$	0	10	7.04	2.90	10	1
How many seminars, conferences and other activities are arranged by City University relating Sustainability?	Scale based	$S_{13}$	0	10	4.57	3.18	10	0
Are you aware how many research-based activities/papers City University has published relating Sustainability till date?	Scale based	$S_{14}$	0	10	4.54	3.24	10	0
On a scale of 0–10 identify the university approach in offering any specialization or training courses regarding sustainability in the campus?	Scale based	$S_{15}$	0	10	4.60	3.14	10	0
In your opinion, how to achieve the goal to have a sustainable campus? 1. Students' intake reduction 2. Green building certification 3. Introducing Curriculum for Sustainability 4. Both 2 & 3 5. Did not answer 6. All of them	Option based	$O_{4}$	1	6	2.27	1.08	5	–

Descriptive analysis is performed to summarize features from the collected dataset. For instance, the minimum, maximum, range, mean and standard deviation are obtained for the demographics of the collected dataset to analyze the overall statics of the received answers. This will help to analyze (1) which departments of the university participated actively in the survey questionnaire, (2) the gender (male/female) involvement in the survey studies (3) to see the participants academic qualification level (undergraduate and graduate), (4) highest education and the semester (only for students) of the respondents. On top of the basic statistical tools, some computational techniques are also performed upon the dataset to analyze the remaining k = 19 queries from a wider perspective. For instance, the collected dataset is fitted to the normal distribution to analyze and examine the fit by using a histogram using MATLAB. The distribution will help to analyze the questionnaire estimates for the mean and standard deviation with the 95% confidence interval. The mean values of the survey questions are used to evaluate the collected responses. Since the scale-based questions have a scale range of 0–10, the mean values for each of the scale-based question is classified into 0 and 1. For example, questions with mean value less than 05 are indicated as 0, whereas the questions with mean value equal to or more than 5 are indicated as 1. The purpose of classifying the mean values into 0 and 1 is to deduce the meaningfulness of the responses. For example, scale-based questions with mean value less than 5 indicate that the respondents have less than 50% knowledge of the specific question, which in turn determines the question with the least information in terms of knowledge, understanding, interest, and so on. The same is true for questions that contain equal to or more than 50% of the information regarding sustainability. We have referred these indications 0 and 1 as flag values, just for reference. As the questionnaire has a few option-based queries so they will be directly assessed based upon their answers. As previously said, the primary goal of the study is to evaluate the collected survey questionnaire and get a comprehension of the sustainability from many aspects inside the HEI in KP province of Pakistan; hence, the analysis of the study is kept simple yet credible.

## 3 Results & discussions

As mentioned in the preceding section that the analysis of the questionnaire is split into two categories, faculty and staff response and (2) Students' response. We will first analyze the faculty and staff response and later assess the students’ responses.

### 3.1 Faculty and staff responses

The statistical analysis for the demographics of the study is tabulate in Table 1. The demographical part of the study includes the questions regarding the (1) Gender of respondent, (2) Position of the respondents (faculty/staff/student), (3) semester (only for students) (4) Educational level of the respondents and (5) The department of the respondent (see Table 1). The (n = 46) responses are fed into the routine of basic statistical analysis, which yielded the minimum, maximum, mean, standard deviation and the range (see Table 1). From Tables 1 and it is clear that the mean value of the gender of the respondent is found to be (1.24) which corresponds to the fact that more males have participated in filling the survey questionnaire. This may raise the question of why male participation statistics are greater than female participation. To answer the question, we will focus primarily on the faculty male to female ratio in each department because it differs by department. For example, the Civil Department has no female faculty members, the Architecture Department has one, the English Department has five, and the Computer Science Department has only three. The information comes from the university's official website. Therefore, we can see more male participants in the survey. The second demographical question is about the position of the respondent, the question is posed to know the respondent is from the faculty/staff. The minimum range of the question is 1 which corresponds to the faculty member and the maximum value is 2 which corresponds to the staff member. A mean value of (1.30) indicates that the most faculty members have participated in the filling the survey questionnaire in contrast to the staff of the university. Again, there might be reasons for the observation. For example, if the staff's burden is too great and they are unable to participate, etc. To answer the question, it is necessary to delve deeper, which is beyond the scope of the current study. The third demographical query is posed to ask the respondents department in the university and when looking at Table 1 it is observed that the minimum and maximum range of the query is 1 and 9 and the mean value of (3.72) indicates that the respondents are mainly from the Social and Natural Science departments. The last demographical query is related to the highest education level earned by the respondents, and it turned out the master's studies is profoundly seen in the collected data set for this particular observation with a mean value of (1.78). In the preceding paragraph, the sustainability related queries are evaluated for the faculty/staff of the university.

**Fig. 1:**
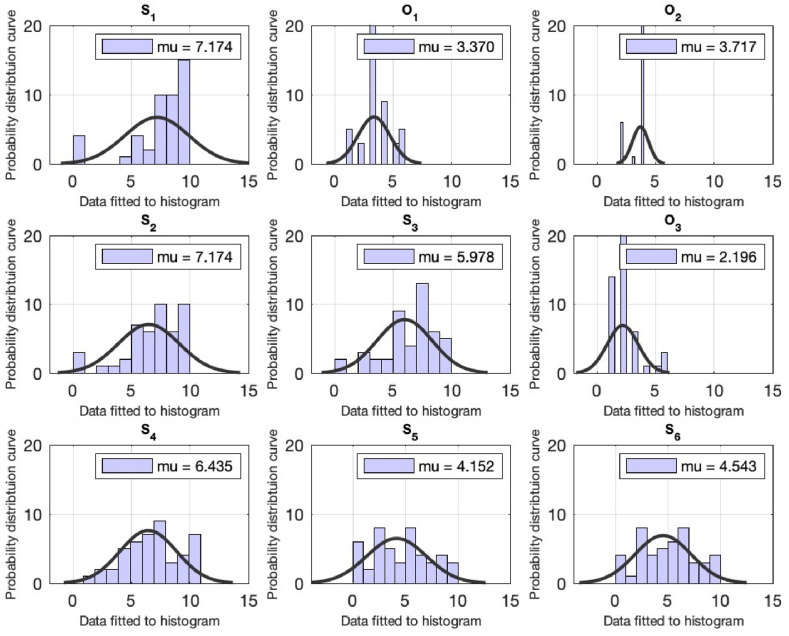
Computational analysis for the queries from $S_{1}$ till $S_{6}$ for the faculty of the university. On the x-axis, the distribution data is fitted to the histogram. The y-axis represents the normal distribution density curve for the received responses.

**Fig. 2:**
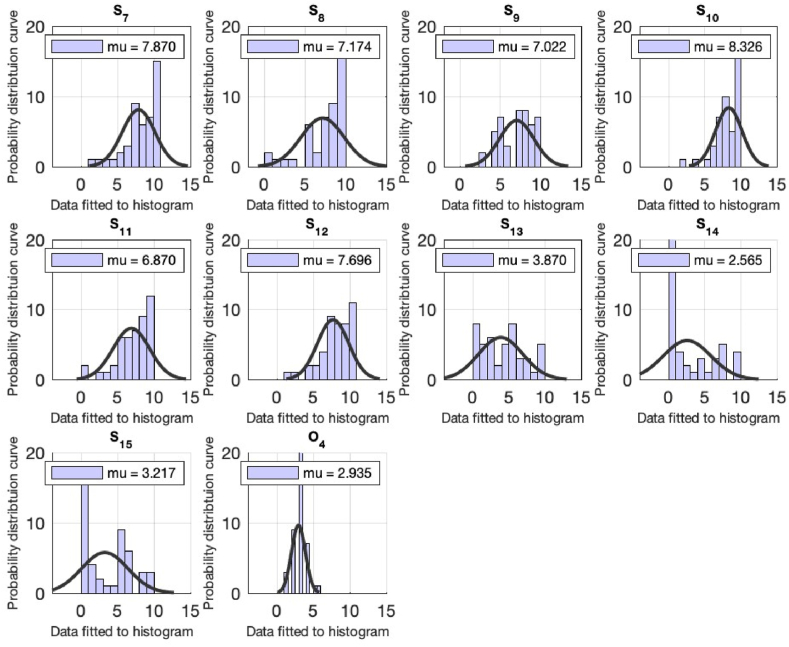
Computational analysis for the queries from $S_{7}$ till $O_{4}$ for the responses. On the x-axis, the distribution data is fitted to the histogram. The y-axis represents the normal distribution density curve for the received responses.

Each of the queries assess individually using the computational techniques via MATLAB. A normal distribution object is created by fitting the data (each query) into it. The distribution object includes the (data) estimates for the mean and standard deviation, and the 95% confidence intervals for the data. Then the histogram is created using the values in data (each question) using the number of bins equal to the square root of the number of elements in data and fits a normal density function see Table 2 and Fig. 1, Fig. 2. The computational technique is performed for the k = 19 queries and the results are tabulated in Table 2 (for the faculty) and the pictorial representation is plotted in Fig. 1, Fig. 2. We will be analyzing the responses for each question in detail in the preceding section.

First three questions of the questionnaire are related to the respondent's knowledge of sustainability (scale based), meaning of sustainability (option based), factors important for sustainability (option based). The statistical analysis yielded average values 7.17, 3.37 and 3.71.

The preceding questions are about the respondent's opinion for the non-technical field's contribution in sustainability, their knowledge about the idea of green buildings and what could have made their university as green campus. For these questions the mean values of 7.17 (scale based), 6.00 (scale based) and 2.10 (option based) are obtained. The respondents are also inquired about their thought of individual practicing the sustainability (scale based), data access for advancing in the field of sustainability (scale based), their knowledge about ranking of Pakistan in the Global Climate Risk Index list and recycling practices can achieve sustainability. The responses which are received are with the mean 6.43, 4.15 and 4.54, 7.47 see Table 2.

The preceding questions are asked from the respondents about their participation and interest sustainability related academic activity, their interest to know more about sustainability, willingness to learn about sustainability and preference level for jobs related sustainability. The corresponding mean values are tabulated in Table 2. The remaining queries of the questionnaire are pertaining to the university approach in offering sustainability related content and the seminars, and the answers of the queries are tabulated in Table 2.

### 3.2 Students’ responses

**Fig. 3:**
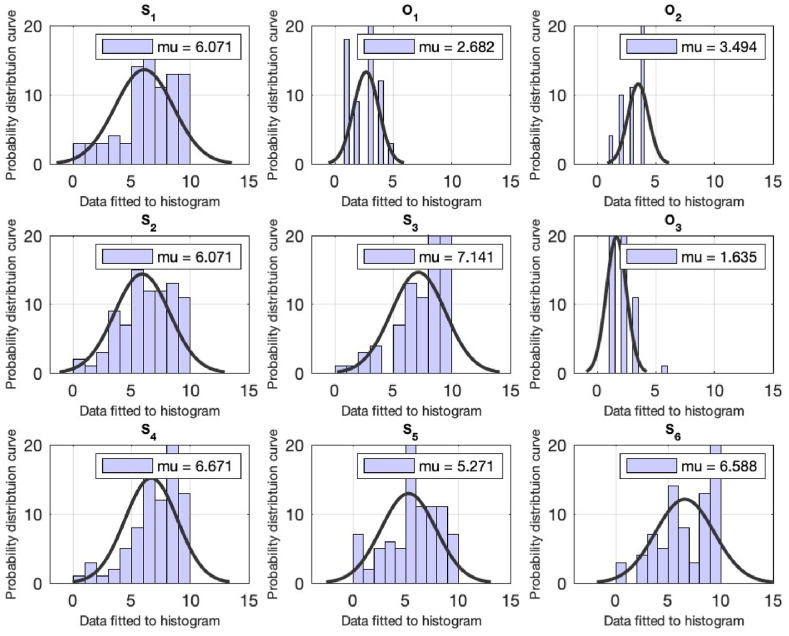
Computational analysis fot the queries from $S_{1}$ till $S_{6}$ for the student of the university. On the x-axis, the distribution data is fitted to the histogram. The y-axis represents the normal distribution density curve for the received responses.

**Fig. 4:**
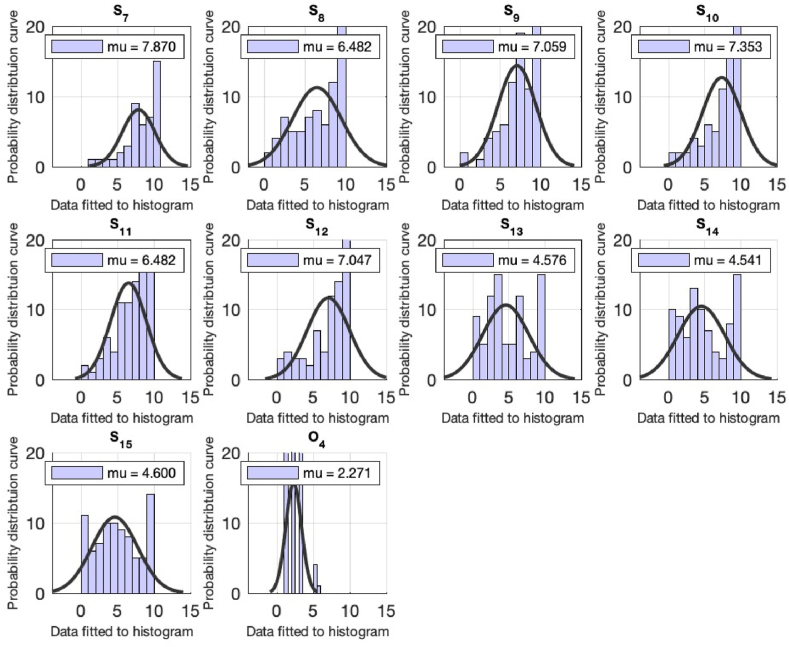
Computational analysis fot the queries from $S_{7}$ till $O_{4}$ for the student of the university. On the x-axis, the distribution data is fitted to the histogram. The y-axis represents the normal distribution density curve for the received responses.

A total of n = 131 responses have been received from the students. The statistical analysis for the demographics of the study is tabulate in Table 3 and depicted in Fig. 3. The demographical part of the study includes the questions from students regarding the (1) Gender, (2) Position of the respondents, (faculty/staff/student), (3) Semester (4) Educational level of the respondents and (5) The department of the respondent (see Table 4). From Table 2, the mean value to each of the demographical queries are 1.12, 3, 2.85, 5.22 & 1.08 which indicates that (1) more males have participated in survey, (2) mainly the 5th and 6th semester students actively participated, (3) the most students are form the under graduation and (4) mainly from the engineering department. The next section provides the statistical analysis of the sustainability related queries (see Fig. 4).

The first question in Table 4 is addressed to assess students' knowledge of sustainability, and the mean result of 6.04 suggests that students do understand sustainability. To validate the previous question, students are asked to define sustainability by selecting one of four options: increase/decrease in energy demand and increase/decrease in population. With a mean value of 2.68, the majority of students perceived sustainability as an increase in energy demand. The next question is to assess student opinion on the variables that are vital for sustainability. This is another option-based question, and many students picked the social, economic, and environmental aspects to be important for sustainability with a mean value of 4 see Table 4 & Fig. 4.

When the students are urged to indicate their opinion on the contribution of non-engineering fields to sustainability, the mean value of 6.07 indicates that the students agreed that non-engineering fields could also contribute to the sustainability of the campus. In addition, a general question from the students about the awareness of green buildings is asked, which shows that around 70% of students understand the idea of green buildings. An option-based question is posed to ask students about making university a sustainable campus, and the respondents think to achieve sustainability at the campus is through the adoption of reuse, reduction, and recycling activities with the mean value of 1.63 see Table 4.

Students have agreed with the statement that individual practicing sustainability can made a difference (mean of 6.67). When the students were asked about their opinion on the claim that access to data access for advancement in the field of sustainability is not relevant, very few of the students agreed with the assertion (5.27). The next question concerns the awareness of the respondent on Pakistan's current ranking in the Global Climate Risk Index, and a satisfactory result of mean 6.58 indicates that students have an idea of Pakistan ‘s ranking in the Global Climate Risk Index. When respondents are asked to agree that recycle, reduce and reuse activities will help to attain sustainability, on a scale of 7.87 the response indicates that the respondents agreed with the argument.

Another question is inquired about the involvement of students in lectures, conferences, workshops on sustainability and a mean value of 6.48 indicates a significant involvement. When students are asked about their level of interest in reading and (or) listening to news related to sustainability and willingness to learn about sustainability, mean of 7.05 and 7.35 indicates a satisfactory answer. When the student's choice for work is examined if income is not a consideration and the wastewater recycling demand is pursued and sold to the local community at subsidized rates, mean values of 6.48 and 7.04 are obtained.

The few queries are inquired about the university approach in arranging the sustainability related activities, research-based publications, and university approach in offering specialization courses on sustainability. For all these queries the mean value of less than 5 is attained (see Table 4).

The last question is the option-based question and is inquired about the respondents opinion on the goals to have a sustainable campus for which the students have selected the following; Students’ intake reduction, green building certification and introducing curriculum for sustainability can, all can help to have a green campus (see Table 4).

Sustainability in the university requires input from multidimensional areas such as social, economic, and environmental, which in turn includes various factors such as understanding, behavior, and attitude of students and faculty towards sustainability, which is currently the subject of research. The bottom line in all research studies stays the same—the single goal of being and acting sustainably. This research study is indeed one of the study approaches to find out the various factors relating sustainability from the student and the faculty perspectives. The analysis of the study is descriptive, and the answers are drawn based upon the scale based and the option-based questions. As mentioned earlier, for each of the scale-based question, the mean value is obtained first, if the mean value is less than 5, the question is allotted the indication of 0 and if the mean value of the query is equal or greater than 5, then the question is allotted an indication of 1. We have referred these indication values as flag values. From this point onwards, we will evaluate the results based on these flags values.

Initial questions of the questionnaire are mainly related to the respondents' knowledge, understating and the information relating the sustainability. The statistical analysis yielded that both the faculty and students have indicated a flag value of 1 for the scale-based questions. This included the questions with queries labelled as ($S_{1}-S_{7}$) see Table 2, Table 3. There is an exception for the faculty response with query labelled as $S_{6}$. This question is about the respondents’ response about the knowledge of Pakistan ranking in Global Climate Risk Index and the flag value is 0 which is indicating that the faculty members are least knowledgeable of the Pakistan ranking in Global Climate Risk Index.

One of the option-based question (labelled as $O_{1}$ both in Table 2, Table 4) it is inquired from the respondents about their meaning of sustainability by providing the four options. The response of the query seems to be dwindling between the increase in energy demand and decrease in energy demand. According to the explanations provided in Refs. [30,31], the rise in population raises both the demand for available natural resources and energy demand. The natural resources used to support the population are limited. Therefore, an increase in population causes an increase in energy demand which results in the use of limited natural resources and therefore results in an unsustainable system. Also, under the traditional definition of sustainability, ‘Sustainability focuses on meeting the needs of the present without compromising the ability of future generations to fulfill their needs. Understanding the definition of sustainability is therefore important prior to its implementation [32]. As a result, it is deduced that the concept of sustainability must be effectively communicated to the respondents.

Another option-based query is asked from the respondents (labelled as $O_{2}$) regarding the essential elements for sustainability (social, economic & environmental) and the respondents have selected all of the factors as important for sustainability. This indeed is true that all the social, economic and environment factors are important for sustainability.

The next few queries are scale-based queries and are related to the respondents' interest in the sustainability related activities. This includes all the question labelled as $S_{8}-S_{12}$ see Table 2, Table 4. The flag value of 1 is observed for all these scale-based questions for both faculty and students’ response. Based on the flag value of 1, it can be inferred that the respondents are interested in content relating sustainability.

The last few queries (labelled as $S_{13}-S_{15}$) are all related to the university approach in organizing or conducting the sustainability related seminars, conferences, university approach in publishing the sustainability related content and university approach in offering any specialization or training courses regarding sustainability. Both the faculty and students have indicated the flag value of 0 for these queries. According to the statistics of this study, there appeared to be a limitation of university commitment, knowledge transfer from the institution, and resources to establishing a platform for promoting sustainability. In one of the research study, it is stated that consistent with the fact that the University's commitment to green thinking is also one of the key factors in sustainability practices, but only commitments will be insignificant if the individual as well as the entire body of the institution are not optimized [24,33]. As a result, an essential factor of university sustainability is absent in terms of institution commitments to think green. The findings of this study reveal that a commitment from the university is required to ensure the campus sustainability.

HEI's are the major contributor to the energy sector in any country setting [[34], [35], [36]]. It is important to mention a few of the energy initiative that can be taken to make the energy sector sustainable from the perspective of HEI's contributions and possible practices that could be taken within the campus that can lead to a positive impact on the energy sector. A few possible contributions will be as follows.

Characterizing the energy related content into the emissions, campus electrification, behavioral change through the being green practices, energy conservation and energy efficiency practices, recycling and reducing practices etc. can be adapted to contribute to the energy sector. For example, because the institution uses energy for heating and cooling, the quantity of carbon emissions may be quantified and monitored in order to mitigate it. Likewise, it is a better course of action to establish renewable energy capacity through utilities, which can offset the carbon emissions plan. The campus building can be designed, renovated, or restructured in accordance with the energy conservation strategy plan. The reward practices in being green may also be a particularly beneficial effort. For example, incentives can be granted departmentally, and point systems can be implemented to attain the green title.

Since the practices of contributing to the energy sector are so diverse, they are always characterized by the domain of the research area and a multitude of the other variables involved. As a result, the few approaches mentioned in the preceding paragraph, as well as other more criterions and techniques, might be adopted and executed by HEI's to contribute to the energy sector.

As already mentioned, there are multifaceted factors based on which the notion of sustainability in the university is assessed. Therefore, the research is being conducted at various HEIs to identify the essential linkages between the various components of university sustainability and this study is one of the efforts and it is identified that the respondents have an interest in the university sustainability but at the same time it is also identified that there is the lack of institutional commitment in providing the suitable platform for the university sustainability. This is one of the case studies from one HEI in Pakistan; more research from different institutions may yield different results; thus, this study alone cannot characterize all the factor important for university sustainability; therefore, much more research is required to dig deeper into university sustainability.

## 4 Conclusion

This research study is one of several initiatives to examine and evaluate sustainability in university by posing various sustainability-related questions to university students and faculty. The study's findings demonstrated that both university students and faculty expressed a strong interest in their knowledge, comprehension, and interests in sustainability. However, the study results revealed that the university has the least dedication to advocating, practicing, and implementing sustainability. There appeared to be a lack of dedication on the part of the university. The research's findings are purely descriptive in nature, with no comparisons made between faculty and student responses because comparing responses was beyond the scope of this study. As previously said, this case study is one of the many approaches for determining different factors associated to sustainability by surveying university students and faculty. The study's findings, although simple in nature, but are reliable and can serve as baseline data for examining sustainability in HEI. This study may potentially give additional perspectives while analyzing sustainability in HEI.

## 5 Limitations

The study highlighted a wide range of factors associated with sustainability in the HEI. A survey-based response is gathered from the university students as well as the faculty. The survey questions in their current form are quite fundamental and basic in nature, yet they provide an insight by understanding the sustainability fundamentals inside the university. However, the study does have certain limitations. For example, the study is a typical case study from one of the HEI in the region. Extending the research area from one to several HEIs from the same and different regions would undoubtedly give a more comprehensive and thorough view on HEI sustainability. Furthermore, the research questions are not classified under the umbrella of the social, economic, and environmental perspectives, demonstrating the general overview of sustainability in universities. If the research questions are classified according social, economic, and environmental perspectives, a highly thorough image of the university's sustainability will be generated. Also, it has been observed that there is a lack of commitment from the institution in providing resources for HEI sustainable initiatives, but what causes this lack of commitment? Is still another question that must be addressed.

## Funding sources

This research received no specific grant from public, commercial, or non-profit funding agencies.

## Author contribution statement

Naseha Wafa Qammar: Conceived and designed the experiments; Performed the experiments; Analyzed and interpreted the data; Wrote the paper.

Zohaib Ur Rehman Afridi: Conceived and designed the experiments.

Shamaima Wafa Qammar: Contributed reagents, materials, analysis tools or data.

## Data availability statement

The data that has been used is confidential.

## Declaration of interest's statement

The authors declare no conflict of interest.

## Additional information

Supplementary content related to this article has been published online at [URL].

## Declaration of competing interest

The authors declare that they have no known competing financial interests or personal relationships that could have appeared to influence the work reported in this paper.
